# A good life

**DOI:** 10.7554/eLife.00353

**Published:** 2012-12-13

**Authors:** Eve Marder

**Affiliations:** Department of Biology and the Volen National Center for Complex Systems, Brandeis University, Waltham, United Statesmarder@brandeis.edu

**Keywords:** careers in science, grad school, postdoc

## Abstract

Following a career in science involves long hours and hard work, but as **Eve Marder** explains in the first of a series of columns, it can also be extremely rewarding.

Some years ago I had a postdoc who said to me, “I don't want to be like you, I want a life.” This phrase was then a common refrain among young scientists who were struggling with the decision of whether to stay in academic science. But I was truly taken aback that this intelligent and thoughtful young woman felt she could say this to my face because it assumed and presumed so much. I comfort myself with the thought that if I were to see her today, she would have the grace to be embarrassed to have used those words. I hope that by now she has understood that every individual finds satisfaction in different ways, and that what is a ‘good life’ for one person may not suit another. For example, some of my best friends have splendid gardens that give them great joy. I love looking at other people's gardens, but it would never cross my mind to leave the city—with its harbour and cafés—to live in a house with a garden.

When I was a child, my father told me that the secret to a good life was tricking the universe into paying you a salary to do what you loved doing. He said that he watched, with great sadness, friends who were aspiring poets, painters and musicians who worked jobs they hated to pay the bills, and then tried to paint and write after they were tired by days filled with tedium. In contrast, I get paid to do a job that I enjoy; I choose to work long hours because I spend most of those hours doing things I value. Family is also central to a good life. Families can be those we were born into, those we create, and those we may find at work. Science often creates strong bonds between labmates and colleagues, and friendships that endure for lifetimes.

What can I say to those of you embarking on careers in academic science at a time in history fraught with anxiety about high-profile papers and grants? Doing science well has never been easy. In the long-gone days of slide rules, my undergraduate thesis advisor told me that the more time I put into doing experiments, the more I would get in return. (He was concerned because I was sharing my time between the lab and various anti-Vietnam War activities.) From time to time I still hear his voice in my head as I say the same to my students.

When I started graduate school in 1969, one of the senior students said to me, “Why are you here, don't you know there will never be a job for you?” I remember just staring at him and saying that I just wanted to be in graduate school. There were times during grad school—and later as a postdoc and an assistant professor—when I thought about quitting. But I didn't, because at the end of day, I liked thinking about science, working in the lab, reading about science, and being around smart people. And I liked my independence and autonomy. So I kept going, often with no more than a vague trust that when one did things for the right reasons, the future would take care of itself.

Much of my day is spent in conversation in my office or in hallways, trying to answer questions like: Should I go to graduate school? Am I ready to apply for a faculty position? What journal should I send this paper to? How do I deal with the stupidity of Reviewer 3? Where are we going to find money for such and such? I never view these conversations as wasted time. But, these conversations cost me “work time”, and they are part of the reason I find myself working the long hours that prompted my then postdoc to say she didn't want to be like me.

**Figure 1:**
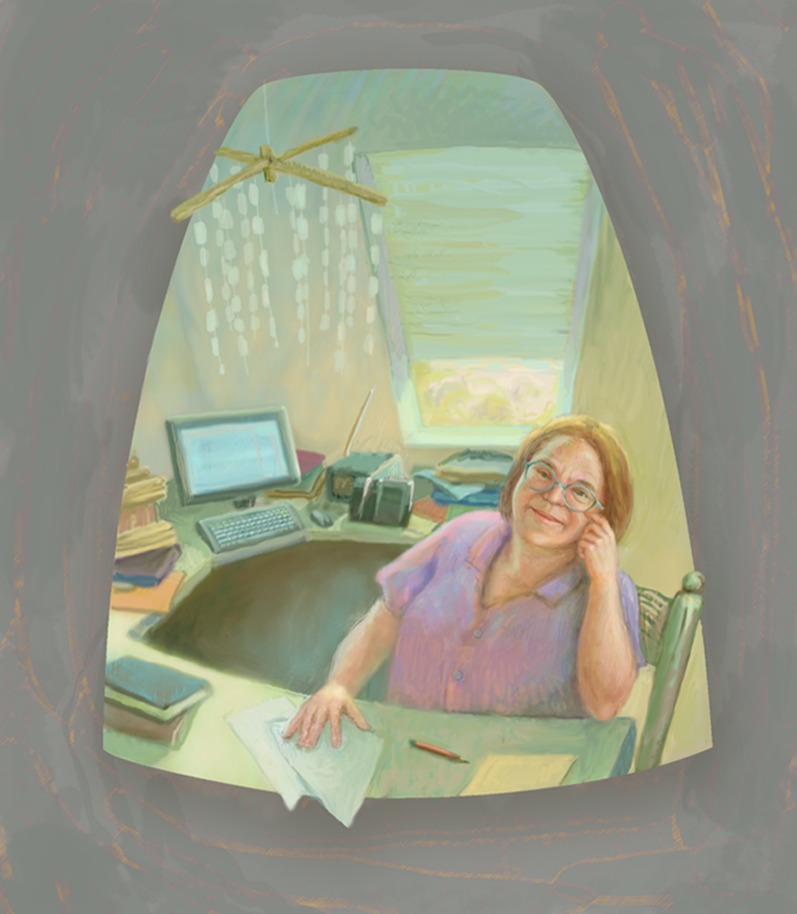
This illustration of Eve Marder in her office at Brandeis was drawn by her nephew Ben Marder.

ILLUSTRATION: BEN MARDER

The answer to my ex-postdoc is that I work long hours because I find work interesting. And yes, papers do get rejected, and not all grant applications get funded, and not all students are equally brilliant, and I do go to too many stupid meetings, and yes there is much wrong with the way we do science today that needs fixing. But, there remains something quite magical about a life that allows me to work with students from whom I learn, and with whom I can make small discoveries that touch on some of life's deepest secrets, and that takes me to conferences where I encounter people who sparkle with intellect, insight and humour.

Some of you are probably saying to yourselves: “It's all very well and good for her, because she has a job at a good institution with a wonderful neuroscience program, but what if I am not recruited by a leading institution and end up at a lesser place?” Outstanding junior recruits have established excellent research programs at many institutions. As I travel around the world, I meet fabulous graduate students everywhere. There are bright and energetic young faculty at every institution I visit, and many of them find it easier to flourish without the elevated expectations that are often present at our most elite institutions. A good job for you will be at an institution that gives you the intellectual space to develop as an independent scientist, and this might be anywhere in the world, on any of the seven continents. There are numerous scientists of international repute who spent their formative years at less-well known institutions. Many of them will tell you that their careers benefitted from the freedom to make their own mistakes in peace, away from the limelight.

A good job for you will be at an institution that gives you the intellectual space to develop as an independent scientist, and this might be anywhere.

Yes, doing science can demand long hours, and there are periods when the rewards seem illusive and elusive. But, it can also be a good life, filled with intellectual vitality and community. For those of you who feel the call of the unknown, and who want to see a small piece of life's mysteries for the first time in human experience, science is a wonderful path through still unknown forests and mountains, although like most hiking trails, the path will dip into mire and fog along the way.

